# Synthesis and quantitative structure–activity relationship study of substituted imidazophosphor ester based tetrazolo[1,5-*b*]pyridazines as antinociceptive/anti-inflammatory agents

**DOI:** 10.3762/bjoc.9.199

**Published:** 2013-08-22

**Authors:** Wafaa M Abdou, Neven A Ganoub, Eman Sabry

**Affiliations:** 1 Chemical Industries Division, National Research Centre, Elbohouth St. D-12311, Dokki, Cairo, Egypt. Fax: 202 7601877

**Keywords:** antinociceptive/anti-inflammatory agents, imidazophosphor esters, phosphonyl carbanions, ring closure, tetrazolo[1,5-*b*]pyridazine

## Abstract

A high-yielding general synthesis of imidazophosphor ester based tetrazolo[1,5-*b*]pyridazines is described. A conjugated reaction between 3,6-diazidopyridazine and different types of phosphonyl carbanion reagents followed by intramolecular cyclization afforded the target products, by using sodium ethanolate solution as a reaction medium. Among the products, five compounds, at a dose of 50 mg per kilogram body weight, showed a notable antinociceptive and anti-inflammatory activity without toxic side-effects.

## Introduction

Inflammation is a characteristic feature of disease pathology and progression in several neuro-degenerative disorders and physical functioning [1–2]. Recently, nonsteroidal anti-inflammatory drugs (NSAIDs) are well established for the treatment of inflammatory disorders [3–5]. The anti-inflammatory effect of NSAIDs is mainly based on the inhibition of the cyclooxygenase (COX) enzymes. Later on, it was reported that the second isoform of cyclooxygenase (COX-2) has a better effect on the inflammation with fewer side-effects [6–9]. Despite their widespread use, none of the presently available agents is ideal; each has its own shortcomings [3]. Subsequently, to improve the efficacy/safety profile of new NSAIDs, the structural–activity relationship (SAR) has been extensively studied, taking into account up-to-date knowledge about the mechanism of inflammation that balanced the inhibition of COX-1, COX-2, and lipoxygenase (LOX) [10–12].

As a part of our continued interest in the development of convenient synthetic approaches to β-enamino- and α-aminophosphonates with anti-inflammatory properties [13–20], we recently successfully synthesized a series of mono- and bisphosphonate-based tetrazolo[1,5-*a*]quinolines with marked anti-inflammatory properties [21–22]. Following this, synthesis of the target compounds, substituted tetrazolo[1,5-*b*]pyridazinphosphor esters, is described herein. In this context, we applied different types of phosphonyl carbanion reagents to 3,6-diazidopyridazine (**1**) as an adopted substrate. The anti-inflammatory and the antinociceptive properties of the prepared compounds were screened and the structure–activity relationships were studied. The anti-inflammatory properties of many tetrazole [21–23] and pyridazine derivatives have also led to their clinical application as NSAIDs (e.g. Bucolome) [24]. Several phosphonate derivatives also exhibit marked potency as inhibitors of COX-1 and COX-2 and are therefore believed to be useful as anti-inflammatory drugs [25–26]. Thus, we considered that it is of interest to gather these three motifs in one molecule.

## Results and Discussion

**Scheme 1:**
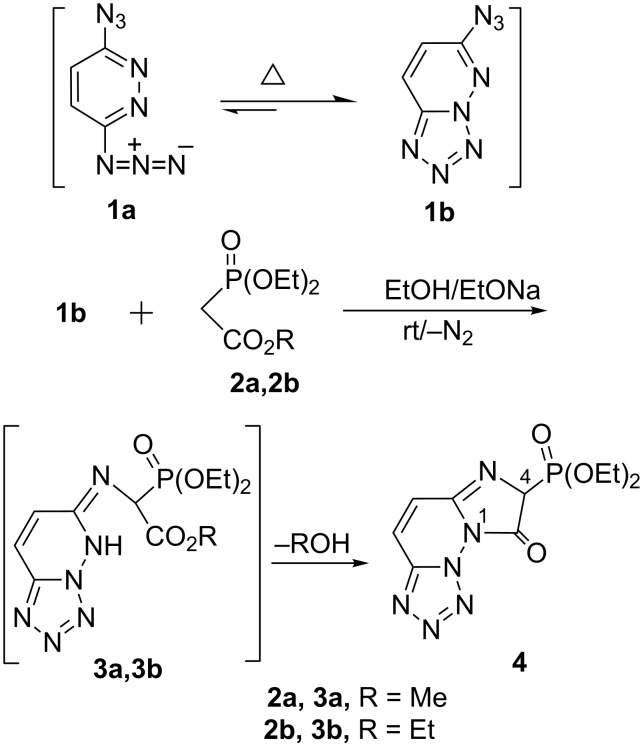
Synthesis of substituted diethyl oxophosphonate **4**.

**Scheme 2:**
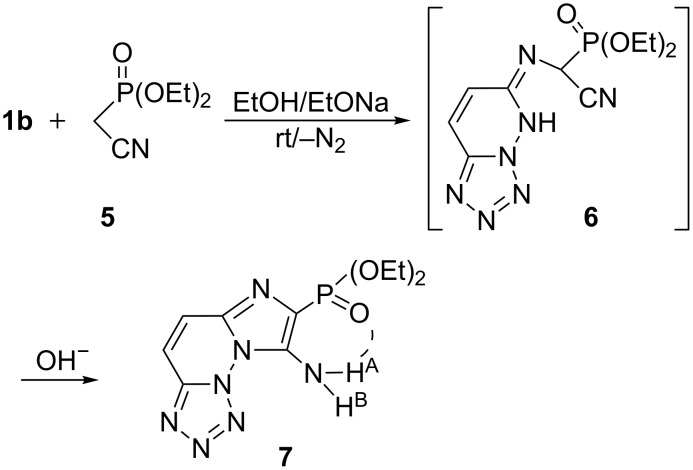
Synthesis of substituted diethyl aminophosphonate **7**.

**Scheme 3:**
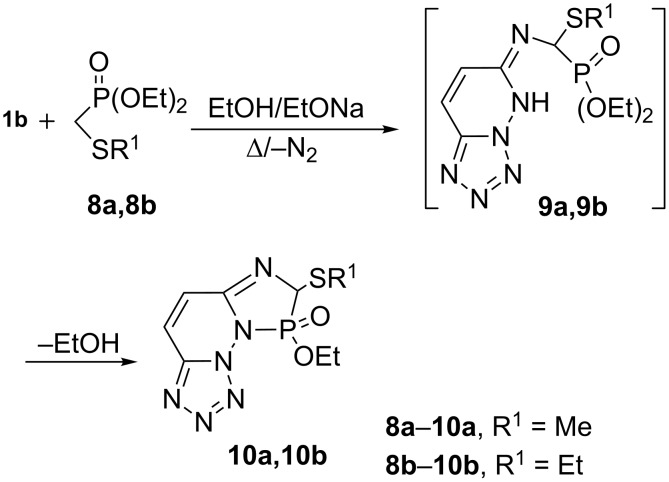
Synthesis of fused diazaphospholo-substituted compounds **10a, 10b**.

**Scheme 4:**
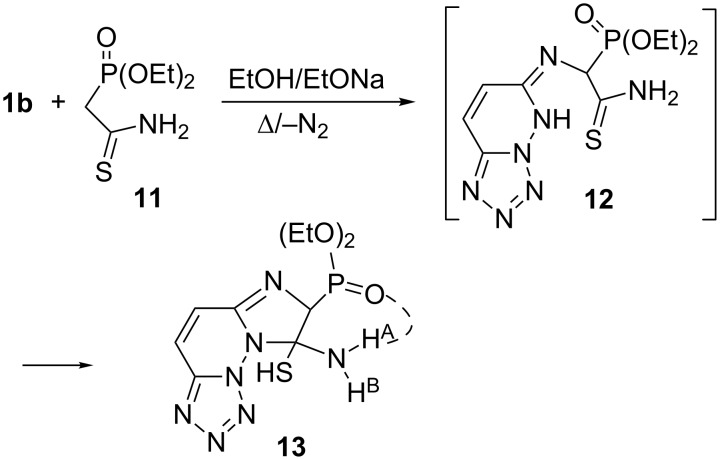
Synthesis of fused imidazophosphono-substituted compound **13**.

**Scheme 5:**
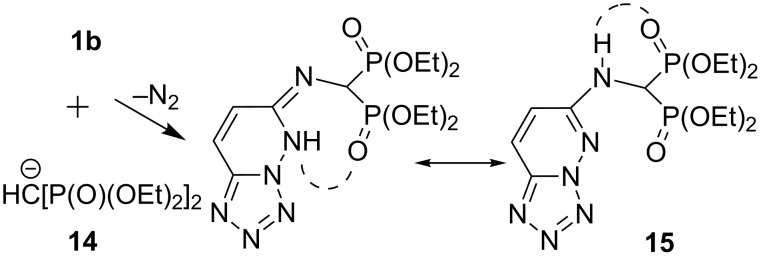
Synthesis of β-enaminobisphosphonate **15**.

**Scheme 6:**
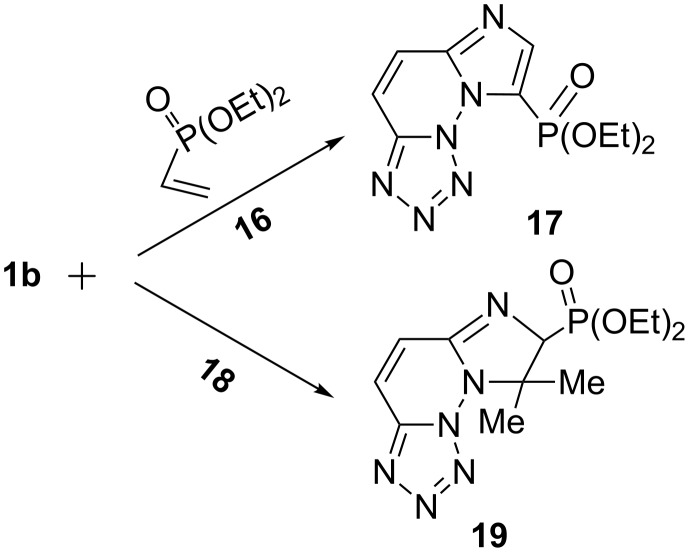
Synthesis of fused imidazophosphono-substituted compounds **17** and **19**.

**Scheme 7:**
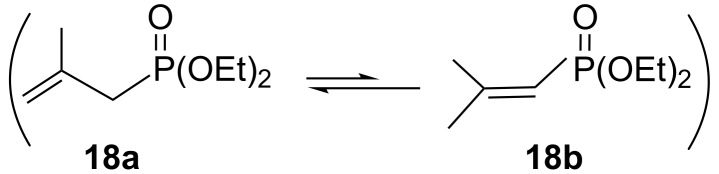
Isomeric forms of diethyl 2-methylallylphosphonate (**18**).

It has been reported that 3,6-diazidopyridazine presents azido (**1a**) 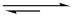 tetrazolo tautomerism (**1b**) whereby it is mainly in the tetrazole form **1b** [27–29]. Accordingly, and as indicated from the spectral data of our products, we considered that the substrate 3,6-diazidopyridazine (**1a**) is exclusively, throughout our investigation, reacted in the tetrazolo-form **1b** [28–29] (Scheme 1). The required [3,6-diazidopyridazine (**1a**) 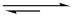 6-azidotetrazolo[1,5-*b*]pyridazine (**1b**)] was prepared by treating the readily available 3,6-dichloropyridazine with sodium azide [30]. The reactions studied and the products obtained are depicted in Schemes 1–7 (see below). Reaction of **1b** with 1.3 equivalents of methyl diethyl phosphonoacetate (**2a**) or the ethyl analogue **2b** in sodium ethanolate solution resulted in the same product assigned as diethyl 8-oxo-7,8-dihydroimidazo[1,2-*f*]tetrazolo[1,5-*b*]pyridazin-7-ylphosphonate (**4**, 78%) as indicated from the analytical and the spectroscopic data.

In the ^1^H NMR spectrum (CDCl_3_) of **4** (δ_P_** =** 28.4 ppm) there is a doublet peak (^2^*J*_P–H_ = 22.3 Hz) at δ 5.36 ppm corresponding to H(4) of the imidazole ring while its C(4)–P appeared at δ_C_ = 59.4 (d, ^1^*J*_P–C_ = 148.6 Hz). A plausible mechanism for the formation of tetrazolopyridazinophosphonate **4** is presented in Scheme 1. Upon heating, the equilibrium between the azido-**1a** and its isomeric tetrazolo-**1b** form [**1a** 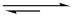 **1b]** lies exclusively at the tetrazole isomer **1b** [29]. Compound **1b** is then intercepted by the nucleophilic attack of the phosphonyl carbanion **2a** or **2b** on the azido-function in **1b** yielding the phosphonate intermediate **3** along with the evolution of a nitrogen molecule. Subsequent intramolecular ring closure, the fused imidazolo-phosphonate **4** would be obtained under elimination of an appropriate alcohol moiety.

In the same fashion, the substrate **1b** reacted with the phosphonyl carbanion, diethyl cyanomethylphosphonate **5**, in ethanolate solution to yield diethyl 8-aminoimidazo[1,2-*f*]tetrazolo[1,5-*b*]pyridazin-7-ylphosphonate (**7**) in 74% yield. The IR spectrum of the phosphonate **7** (δ_P_ **=** 27.8 ppm) showed the NH_2_, P=O, and P–O–C motifs at ν 3377–3330, 1226, and 1123 cm^−1^, respectively. Its ^1^H NMR (CDCl_3_) spectrum showed the NH_2_-protons at δ 6.44 (H^A^, br) and 8.88 ppm (H^B^, br), which are attributed to the P=O bonding with one proton of the amino-group. Furthermore, the ^13^C NMR spectrum of **7** revealed, among others, three doublets at: δ 153.6 [d, ^3^*J*_P–C_ = 11.4 Hz, C(2)], 141.2 (d, ^1^*J*_P–C_ = 188.4 Hz, C(4)–P), and at δ 126.4 (d, ^2^*J*_P–C_ = 14.6 Hz, C(5)*–*NH_2_) ppm. As displayed in Scheme 2, the formation of the fused imidazophosphonate **7** was formed via the initial condensation intermediate **6**. Further alkaline hydrolysis of the cyano group and the intramolecular cyclization led to the product **7** (Scheme 2).

Conversely, similar treatment of **1b** with diethyl (methylthioalkyl)phosphonates **8a** and **8b** under the same reaction conditions yielded the fused diazaphospholo-substituted compounds **10a** and **10b** in 72 and 74% yield, respectively. The ^31^P NMR spectrum (CDCl_3_) of the diazaphospholes **10a** and**10b** showed a sharp singlet around 14.5 ppm, which is within the range expected for the assigned structure [31].

A mechanism for the formation of the heterophosphole structure **10** can be rationalized as in Scheme 3 through the condensation of **1b** with **8a** or **8b** to elaborate the intermediates **9a** or **9b** accompanied by the elimination of a N_2_ molecule. Further intramolecular cyclization of **9** afforded the diazaphospholes **10a** and **10b,** respectively, through the loss of an ethanol molecule [31] (Scheme 3).

Next, the fused imidazophosphono-substituted compound **13** (68% yield) was obtained from the reaction of **1b** with diethyl (2-amino-2-thioxoethyl)phosphonate (**11**) in ethanolate solution. Obviously, **13** resulted in the same manner from the intermediate **12** initially formed, as outlined in Scheme 4.

Further, the azidotetrazole **1b** was allowed to react with the Horner–Emmons (HE) reactant, tetraethyl methylenebis(phosphonate) (**14**) under the same reaction conditions to give the respective β-enaminobisphosphonate **15** (≈73% yield). The gem-diphosphonate structure **15** was delineated from IR, NMR and MS spectra. The IR absorptions for **15** showed the 2 P=O stretching frequencies as two bands at 1262 (P=O, free) and 1226 (P=O, bonded) cm^−1^, which could be explained by a preferred conformation of intramolecular hydrogen bonding between the NH proton and one of the P=O moieties. The ^31^P NMR spectrum (CDCl_3_) of **15** showed the presence of two separate doublets with equal ^2^*J*_P–P_ coupling constants 28.4 Hz at δ 25.6 and 24.8 ppm. The ^1^H and ^13^C NMR data were also in accordance with the assigned structure (see Supporting Information File 1). The formation of **15** can be rationalized as occurring in Scheme 5 through the addition of **14** to the azido group with concomitant evolution of a molecule of N_2_ (Scheme 5). Bisphosphonates belong to an important class of BP-drugs used for the treatment of bone diseases such as osteoporosis, hypocalcemia, inflammation and rheumatoid arthritis [32–33].

Finally, we studied the behavior of the azidotetrazole **1b** toward the unsaturated HE reactant, diethyl vinylphosphonate (**16**), and diethyl 2-methylallylphosphonate (**18**) in EtONa solution to give the fused imidazophosphono-substituted compounds **17** (76%) and **19** (74%). Obviously, according to Scheme 6, the coupling cyclization reaction of **1b** with **16** and auto-oxidation resulted in formation of the final product **17** in one step. This behavior is familiar in similar reactions. On the other hand, we presume that the allyl reagent **18**, which could be present in the isomeric forms **18a** 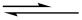 **18b** (Scheme 7), had been conjugated in **18b** form with **1b** to give the phosphonate **19**.

In summary, it has been found that the substrate 3,6-diazidopyridazine reacts with nucleophilic phosphorus reagents, HE reactants, mainly in the tetrazole-form leading to the formation of tetrazolopyridazino-imidazophosphor esters or β-enaminophosphor esters.

## Biological assays

Based on previous reports [24–26] that recognized the pyridazine nucleus is being suitable for anti-inflammatory and antinociceptive agents, and by the fact that ring-fused heterocycles containing more than one nitrogen atom (e.g., tetrazole nuclei [21–23]) are key structures in a large variety of biochemical processes, bioscreening of the synthesized substituted tetrazolo[1,5-*b*]pyridazine-phosphor derivatives was carried out. Thus, keeping the tetrazolopyridazine core structure intact, we studied the effect of different phosphorus-containing moieties on their antinociceptive and anti-inflammatory effects. Substrate **1** was also tested to reflect the effect of its transformations to our products.

### Antinociceptive evaluation *para*-Benzoquinone (*p*-BQ)-induced writhing test

The evaluation of antinociceptive activity of the synthesized compounds was assessed in vivo in mice by using the *p*-benzoquinone-induced writhing test [26]. Ibuprofen was used as a positive control in our experiments; the antinociceptive capacity was expressed as the percentage change compared to writhing controls. The results shown in Table 1 indicated that β-enaminobisphosphonate **15** is the most potent antinociceptive structure (86.4%), which was followed by α-aminophosphonates **13** (84%) and **7** (81%). Indeed, while the azido substrate **1** showed only a weak antinociceptive effect (14%), the eight phosphor compounds demonstrated higher capacity than the reference ibuprofen drug (69.9%) at the same dose of 50 mg per kilogram body weight.

**Table 1:** Antinociceptive/anti-inflammatory effects of the tested compounds on the *p-*BQ-induced abdominal constriction test and carrageenan (CG)-induced hind paw edema model in mice.^a^

tested compound	no. of writhings ± SEM (antinociceptive effect, %^b^)	Swelling in thickness (× 10^−2^ mm) ± SEM (inhibition of edema, %)^c^				anti-inflammatory activity after 360 min, %

		90 min	180 min	270 min	360 min	

control	28.6 ± 2.4	41.4 ± 3.3	55.2 ± 2.7	74.8 ± 3.6	78.2 ± 4.1	—
**4**^d^	6.8 ± 2.2 (76.2)*	30.2 ± 3.8 (27.1)*	28.9 ± 4.6 (47.6)**	37.4 ± 5.7 (50.0)**	31.3 ± 5.6 (60)***	(96.8)**
**7**^d^	5.4 ± 4.3 (81.1)**	31.8 ± 5.3 (23.2)*	25.1 ± 5.6 (54.5)*	33.8 ± 3.4 (54.8)***	27.4 ± 6.4 (65.0)*	104.8
**10a**^d^	5.8 ± 2.9 (79.7)***	30.6 ± 6.3 (26.1)**	26.3 ± 5.6 (52.4)**	34.6 ± 3.6 (53.7)***	27.9 ± 4.3 (64.3)***	103.7
**10b**^d^	6.3 ± 2.9 (77.9)*	30.8 ± 3.8 (25.6)*	27.1 ± 4.7 (50.9)*	35.6 ± 6.0 (52.4)***	28.8 ± 4.8 (63.2)***	102
**13**^d^	4.4 ± 2.2 (84.6)*	30.7 ± 3.3 (25.8)**	23.4 ± 5.6 (57.6)**	33.2 ± 3.6 (55.6)***	25.8 ± 4.3 (67.0)***	108
**15**^d^	3.9 ± 2.1 (86.4)**	28.9 ± 5.2 (30.2)***	21.6 ± 7.4 (60.9)***	28.2 ± 5.0 (62.3)***	22.8 ± 6.7 (70.8)***	114
**17**^d^	7.6 ± 1.7 (73.4)***	30.4 ± 7.2 (26.5)***	29.3 ± 4.1 (46.9)**	39.1 ± 4.2 (47.7)***	30.7 ± 4.5 (60.7)***	97.9
**19**^d^	7.2 ± 2.1 (74.8)***	29.7 ± 4.6 (28.0)*	28.7 ± 5.6 (48.0)**	38.5 ± 5.2 (48.6)***	31.9 ± 3.8 (59.2)***	95.5
**1**	24.6 ± 2.3 (14.0)**	36.8 ± 6.9 (11.1)**	44.6 ± 5.4 (19.2)**	56.8 ± 5.5 (24.0)**	62.4 ± 3.8 (20.2)***	32.6
ibuprofen	8.7 ± 3.1 (69.6)***					
indomethacin		34.6 ± 4.6 (16.4)**	32.4 ± 4.4 (41.3)*	31.4 ± 1.6 (58.02)***	29.8 ± 3.4 (62.0)***	100

^a^Data obtained from animal experiments are expressed as means ± SEM (dose = 50 mg per kilogram body weight, administered subcutaneously to mice (*n* = 6–8). ^b^*p*-BQ-induced writhing; ^c^CG-induced paw edema tests, respectively. ^d^Statistical significance was evaluated from the control by one-way ANOVA post hoc Dunnett’s test (*p < 0.05, **p < 0.01, ***p < 0.001).

### Anti-inflammatory screening

#### Carrageenan-induced hind paw edema test

**Figure 1:**
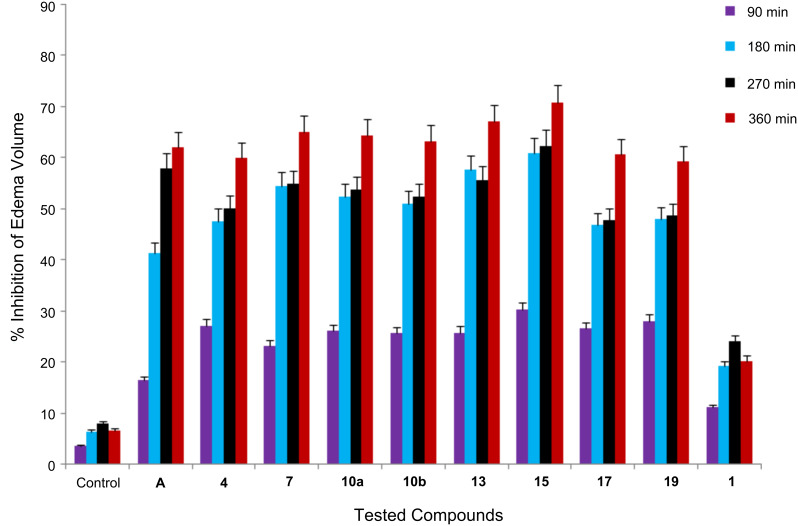
Percentage inhibition of granuloma for the tested compounds at a dose of 50 mg per kilogram body weight after the given time intervals. Error bar: 5%.

Anti-inflammation properties of the new tetrazolo[1,5-*b*]pyridazines-bearing mono- (**4**, **7**, **10a**, **10b**, **13**, **17**, **19**) and diphosphonate nuclei (**15**) were evaluated in an animal model, by the carrageenan–induced paw edema (CPE) method. Following the standard procedures [34–35], these compounds were administered subcutaneously by using 50 mg per kilogram body weight, and the anti-inflammatory effect was measured at successive time intervals (90, 180, 270, and 360 min, after carrageenan injection). The results are profiled in Table 1 and are compared to the substrate **1** and to indomethacin (**A**). β-Enaminobisphosphonate **15** showed the most potent inflammatory properties (e.g., 114% after 360 min) relative to indomethacin. Nevertheless, other compounds displayed good to excellent effects (95 to 108%) in inflammation inhibition after 360 min comparing to **A** without toxic effects. Percentage inhibition of granuloma for the tested compounds at a dose of 50 mg per kilogram body weight at successive intervals is displayed in Figure 1.

According to the results of the biological assay in Table 1, we could deduce the structure–activity relationship (SAR) as follows: (1) among the tested compounds, β-enaminobisphosphonate **15** has the most antinociceptive/anti-inflammatory activity, even higher than the references ibuprofen and indomethacin; (2) there is a parallel correlation between the anti-inflammatory activities and the antinociceptive activity results (Table 1); (3) the amino group substituent has a positive effect (see **7** and **13**); (4) like indomethacin, the tested phosphonates showed gradual increase in the second phase (after 270 min).

#### LD_50_ of the most promising products

In an acute-toxicity experiment, the most promising anti-inflammatory compounds **15**, **7**, and **13** were tested using the LD_50_ standard method in mice at doses of 500, 750 and 1000 mg per kilogram body weight, which is 10–20 times the used anti-inflammatory effective dose (50 mg per kilogram body weight). The assay did not show toxic symptoms or mortality rates throughout the following 24 h post-administration, indicating the safety of the used doses.

## Conclusion

In summary, we have offered a practical and efficient procedure for the synthesis of imidazophosphor esters based tetrazolo[1,5-*b*]pyridazine in high yields by application of different types of Horner–Emmons (HE) reagents on 3,6-diazidopyridazine. Among the products, the β-enaminobisphosphonate compound demonstrated the highest antinociceptive and the anti-inflammatory activities.

## Experimental

See Supporting Information File 1 for full experimental data

## Supporting Information

The experimental section, the general procedures, the experimental data, the results of the analyses, and the bioassay procedures are included in Supporting Information File 1.

File 1 Full experimental details.
