# Supplementary material for: Synthesis and quantitative structure–activity relationship study of substituted imidazophosphor ester based tetrazolo[1,5-b]pyridazines as antinociceptive/anti-inflammatory agents
Source: Beilstein J Org Chem. 2013 Aug 22;9:1730–6. doi: 10.3762/bjoc.9.199 (PMC3778331; doi:10.3762/bjoc.9.199)
Supplement: File 1 — Full experimental details. [file Beilstein_J_Org_Chem-09-1730-s001.pdf]

# Supporting Information

for

# Synthesis and quantitative structure–activity relationship study of substituted imidazophosphor ester based tetrazolo[1,5-*b*]pyridazines as anti-nociceptive/anti-inflammatory agents

Wafaa M. Abdou*, Neven A. Ganoub and Eman Sabry

Address: Chemical Industries Division, National Research Centre, Elbohouth St. D-12311, Dokki, Cairo, Egypt. Fax: 202 7601877

Email: Wafaa M. Abdou* - wabdou@link.net

*Corresponding author

## Full experimental details

## Experimental

Melting points (uncorrected) were determined with open capillary tube on an Electrothermal (variable heater) melting point apparatus. IR spectra were recorded on a JASCO FT-IR 6100 using KBr disc. NMR spectra were measured with a JEOL E.C.A-500 MHz ($^{1}H$: 500.7 MHz, $^{13}C$: 125.4 MHz, $^{31}P$: 200.7 MHz) spectrometer. $^{31}P$ NMR spectra were recorded with $H_3PO_4$ (85%) as external reference. $^{1}H$ and $^{13}C$ NMR spectra were recorded with trimethylsilane as internal standard in $CDCl_3$. Chemical shifts (δ) are given in ppm. The mass spectra were recorded at 70 eV on an MS-50 Kratos (A.E.I.) spectrometer provided with a data system. The appropriate precautions in handling moisture-sensitive compounds were observed. The purity of all new samples was verified by microchemical analysis (H/C/N/P/S) and spectroscopy. Solvents were dried by standard techniques. TLC: Merck 0.2 mm silica gel 60 F254 analytic aluminum plates. All international principles and local regulations concerning the care and use of laboratory animals were considered during the pharmacological screening. Diazide substrate **1** was prepared according to the reported method [1].

## Synthesis

**Reaction of the diazide 1 with phosphonyl carbanions 2a, 2b, 5, 8a, 8b, 11, 14, 16 and 18**

**General procedure:** A solution of 1.3 mmol of phosphonyl carbanion (Horner–Emmons, HE) reagent **2a, 2b, 5, 8a, 8b, 11, 14, 16** or **18** in 10 mL of absolute ethanol containing 2.3 mmol sodium (Na) was stirred at 0 °C for about 30 min. Thereafter, a solution of 1 mmol of 3,6-diazidopyridazine (**1)** (1.6 g) in 10 mL of EtOH was then added in one portion, and the reaction mixture was further stirred at rt. After the completion of the reaction (TLC), the product mixture was cooled, poured into ice-water, and acidified with conc. HCl to pH ≈ 6, followed by extraction with AcOEt (3 × 50 mL), and the combined organic phase was dried over anh. $Na_2SO_4$. After removal of the volatile materials under vacuum, the resulting residue was crystallized from the proper solvent to give the corresponding product **4**, **7**, **10a**, **10b**, **13**, **15**, **17** or **19**.

**Reaction of 1 with 2a, 2b:** Reagents: diazide **1** (1.6 g, 1 mmol), methyl diethyl phosphonoacetate (**2a**, 2.7 g, 1.3 mmol) or triethyl phosphonoacetate, (**2b**, 2.9 g, 1.3 mmol), and 53 mg of Na (2.3 mmol) in 20 mL of EtOH. The reaction mixture was further stirred at rt for 12 h to give the same product compound **4**.

**Diethyl 8-oxo-7,8-dihydroimidazo[1,2-*f*]tetrazolo[1,5-*b*]pyridazin-7-ylphosphonate (4):** Yield 2.4 g (78%); mp 105–107 °C (from $CH_2Cl_2$); $^1$H NMR (500.7 MHz, $CDCl_3$) $\delta_H$ 1.49 (dt, $^3J_{H\text{-}H}$ = 6.5 Hz, $^4J_{P\text{-}H}$ = 4.3 Hz, 2 × 3H, 2$H_3$CCOP), 4.55 (dq, $^3J_{H\text{-}H}$ = 6.5 Hz, $^3J_{P\text{-}H}$ = 5.9 Hz, 2 × 2H, 2$H_2$COP), 5.36 (d, $^2J_{P\text{-}H}$ = 22.3 Hz, 1H, *H*C-P), 7.43, 7.97 (2d, $^3J_{H\text{-}H}$ = 9.4 Hz, 2 × 1H, 2*H*-(4,5)pyridazine); $^{13}$C NMR (125.4 MHz, $CDCl_3$) $\delta_C$ 163.8 (d, $^2J_{P\text{-}C}$ = 9.8 Hz, *C*(O)), 147.8 (d, $^3J_{P\text{-}C}$ = 8.4 Hz, *C*(2)-imidazole), 134.8 (*C*-tetrazole), 127.1, 126.2 (*C*(4,5)pyridazine), 62.4 (d, $^2J_{P\text{-}C}$ = 8.7 Hz, *C*$H_2$OP), 59.4 (d, $^1J_{P\text{-}C}$ = 148.6 Hz, *C*-P), 16.2 (d, $^3J_{P\text{-}C}$ = 8.6 Hz, *C*$H_3$COP); $^{31}$P NMR (200.7 MHz, $CDCl_3$) $\delta_P$, ppm: 28.4; EI-MS: in *m*/*z* (%): 311 (16) $[M - 1]^+$, 283 (21) $[M - 29\ (H + N_2)]^+$, 269 (33) $[M – 43\ (H + N_3)]^+$, 137 (58) ($P(O)(OEt)_2$), 132 (100) $[M – 180\ (H + N_3 + P(O)(OEt)_2)]^+$, 78 (69) (pyridazine); $\nu_{max}$/cm$^{-1}$: 1705 (C(O)), 1618 (C=N), 1254 (P=O), 1165 (tetrazole), 1081 (P–O–C); Anal. calcd for $C_{10}H_{13}N_6O_4P$ (312.2): C, 38.47; H, 4.20; N, 26.92; P, 9.92; found: C, 38.43; H, 4.13; N, 26.87; P, 9.84.

**Reaction of 1 with 5:** Reagents: diazide **1** (1.6 g, 1 mmol), diethyl cyanomethylphosphonate (**5**, 2.3 g, 1.3 mmol), and Na (53 mg, 2.3 mmol) in 20 mL of EtOH. The reaction mixture was further stirred at rt for 12 h to give, after the usual working up, compound **7**.

**Diethyl 8-aminoimidazo[1,2-*f*]tetrazolo[1,5-*b*]pyridazin-7-ylphosphonate (7):** Yield 2.3 g (74%); mp 173–175 °C (from MeCN); $^1$H NMR (500.7 MHz, $CDCl_3$): $\delta_H$ 1.26 (dt, $^3J_{H\text{-}H}$ = 6.7 Hz, $^4J_{P\text{-}H}$ = 4.7 Hz, 2 × 3H, 2$H_3$CCOP), 4.07 (dq, $^3J_{H\text{-}H}$ = 6.7 Hz, $^3J_{P\text{-}H}$ = 5.7 Hz, 2 × 2H, 2$H_2$COP), 6.44 (br, 1H, $H^A$N), 7.15, 8.22 (2d, $^3J_{H\text{-}H}$ = 9.4 Hz, 2 × 1H, 2*H*(4,5)-pyridazine), 8.88 (br, 1H, $H^B$N); $^{13}$C NMR (125.4 MHz, $CDCl_3$) $\delta_C$ 153.6 (d, $^3J_{P\text{-}C}$ = 11.4 Hz, *C*(2)-imidazole), 141.2 (d, $^1J_{P\text{-}C}$ = 188.4 Hz, *C*-P), 133.1 (*C*-tetrazole), 126.4 (d, $^2J_{P\text{-}C}$ = 14.6 Hz, *C*-$NH_2$), 124.8, 117.4 (*C*(5,4)-pyridazine), 65.3 (d, $^2J_{P\text{-}C}$ = 8.7 Hz, *C*$H_2$OP), 16.3 (d, $^3J_{P\text{-}C}$ = 7.5 Hz, *C*$H_3$COP); $^{31}$P NMR (200.7 MHz, $CDCl_3$) $\delta_P$ 27.8; EI-MS: in *m*/*z* (%): 283 (21) $[M^+ - (28)\ N_2]$, 269 (33) $[M - 42\ (N_3)]^+$, 253 (45) $[M - 58\ (N_3 + NH_2]^+$, 137 (62) ($P(O)(OEt)_2$), 116 (100) $[M - 195\ (N_3 + NH_2 + P(O)(OEt)_2)]^+$, 78 (68) (pyridazine); $\nu_{max}$/cm$^{-1}$: 3377–3330 ($NH_2$), 1598 (C=N), 1226 (P=O, bonded), 1165 (tetrazole), 1123 (P-O-C); Anal. calcd for $C_{10}H_{14}N_7O_3P$ (311.2): C, 38.59; H, 4.53; N, 31.50; P, 9.95; found: C, 39.02; H, 4.47; N, 31.44; P, 10.02.

**Reaction of 1 with 8a, 8b:** Reagents: diazide **1** (1.6 g, 1 mmol), diethyl (methylthiomethyl)phosphonate (**8a**, 2.5 g, 1.3 mmol), or diethyl (methylthioethyl)phosphonate (**8b**, 2.7 g, 1.3 mmol), and 53 mg of Na (2.3 mmol) in 20 mL of EtOH. The reaction mixture was refluxed for 1 h to give, after the usual working up, **10a** and **10b**, respectively.

**8-Ethoxy-7-(methylthio)-7,8-dihydro[1,4,2]diazaphospholo[1,5-*b*]tetrazolo[5,1-*f*]pyridazine-8-oxide (10a)**: Yield 2 g (72%); mp 92–94 °C (from cyclohexane); $^1$H NMR (500.7 MHz, $CDCl_3$) $\delta_H$ 1.21 (dt, $^3J_{H\text{-}H}$ = 6.9 Hz, $^4J_{P\text{-}H}$ = 4.9 Hz, 3H, $H_3$CCOP), 2.17 (d, $^4J_{P\text{-}H}$ = 4.4, 3H, $H_3$C-S), 4.62 (dq, $^3J_{H\text{-}H}$ = 6.9 Hz, $^3J_{P\text{-}H}$ = 5.8 Hz, 2H, $H_2$COP), 5.36 (d, $^2J_{P\text{-}H}$ = 16.4, 1H, *H*C-P), 7.28, 8.07 (2d, $^3J_{H\text{-}H}$ = 9.2 Hz, 2× 1H, 2*H*C(5,4)-pyridazine); $^{13}$C NMR (125.4 MHz, $CDCl_3$) $\delta_C$ 165.5 (d, $^2J_{P\text{-}C}$ = 6.8 Hz, *C*(2)-imidazole), 137.7 (d, $^3J_{P\text{-}C}$ = 7.6 Hz, *C*-tetrazole), 129.5, 126.8 (*C*(5,4)-pyridazine), 63.4 (d, $^2J_{P\text{-}C}$ = 10.5 Hz, *C*$H_2$OP), 61.6 (d, $^1J_{P\text{-}C}$ = 145 Hz, *C*-P), 19.1 (d, $^3J_{P\text{-}C}$ = 7.2 Hz, *C*$H_3$COP), 17.7 (d, $^3J_{P\text{-}C}$ = 7.6 Hz, *C*$H_3$-S); $^{31}$P NMR (200.7 MHz, $CDCl_3$): $\delta_P$ 14.8; EI-MS: in *m*/*z* (%): 285 (14) $[M-1]^+$, 257 (18) $[M-29\ (H+N_2)]^+$, 243 (42) $[M-43\ (H+N_3)]^+$, 196 (100) $[M-90\ (H+N_3+SMe)]^+$, 104 (86) $[M^+-182\ (H+N_3+SMe+P(O)(OEt)]^+$, 78 (73) (pyridazine); $\nu_{max}$/cm$^{-1}$: 1610 (C=N), 1263 (P=O), 1180 (tetrazole), 1084 (P-O-C); Anal. calcd for $C_8H_{11}N_6O_2PS$ (286.3): C, 33.57; H, 3.87; N, 29.36; P, 10.82; S, 11.20; found: C, 33.64; H, 3.83; N, 29.41; P, 10.73; S, 11.28.

**8-Ethoxy-7-(ethylthio)-7,8-dihydro[1,4,2]diazaphospholo[1,5-*b*]tetrazolo[5,1-*f*]pyridazine-8-oxide (10b):** Yield 2.2 g (74 %); mp 83–85 °C (from pentane); $^1$H NMR (500.7 MHz, $CDCl_3$) $\delta_H$ 1.17 (t, $^3J_{H\text{-}H}$ = 6.4 Hz, 3H, $H_3$CCS), 1.23 (dt, $^3J_{H\text{-}H}$ = 6.9 Hz, $^4J_{P\text{-}H}$ = 4.9 Hz, 3H, $H_3$CCOP), 2.46 (q, $^3J_{H\text{-}H}$ = 6.4 Hz, 2H, $H_2$CS), 4.18 (dq, $^3J_{H\text{-}H}$ = 6.9 Hz, $^3J_{P\text{-}H}$ = 6.2 Hz, 2H, $H_2$COP), 5.43 (d, $^2J_{P\text{-}H}$ = 16.3, 1H, *H*C-P), 7.18, 8.08 (2d, $^3J_{H\text{-}H}$ = 9.4 Hz, 2 × 1H, 2*H*(4,5)-pyridazine); $^{13}$C NMR (125.4 MHz, $CDCl_3$) $\delta_C$ 166.4 (d, $^2J_{P\text{-}C}$ = 9.5 Hz, *C*(2)-imidazole), 137.9 (d, $^3J_{P\text{-}C}$ = 7.5 Hz, *C*-tetrazole), 129.6, 126.5 (*C*(4,5)-pyridazine), 63.1 (d, $^2J_{P\text{-}C}$ = 8.5 Hz, *C*$H_2$OP), 61.2 (d, $^1J_{P\text{-}C}$ = 145.6 Hz, *C*-P), 25.5 ( d, $^3J_{P\text{-}C}$ = 7.6 Hz, *C*$H_2$S), 19.3 (d, $^3J_{P\text{-}C}$ = 7.3 Hz, *C*$H_3$COP), 14.3 (*C*$H_3$-$CH_2$S); $^{31}$P NMR (200.7 MHz, $CDCl_3$) $\delta_P$ 14.3; EI-MS: in *m*/*z* (%): 299 (19) $[M-1]^+$, 271 (25) $[M-29\ (H+N_2)]^+$, 257 (62) $[M-43\ (H+N_3)]^+$, 196 (100) $[M-104\ (H+N_3+SEt)]^+$, 104 (77) $[M-196\ (H+N_3+SEt+P(O)(OEt)]^+$, 78 (69) (pyridazine); $\nu_{max}$/cm$^{-1}$: 1612 (C=N), 1262 (P=O), 1178 (tetrazole),

1142 (P-O-C); Anal. calcd for $C_9H_{13}N_6O_2PS$ (300.3): C, 36.00; H, 4.36; N, 27.99; P, 10.32; S, 10.68; found: C, 36.06; H, 4.42; N, 28.44; P, 10.38; S, 10.61.

**Reaction of 1 with 11:** Reagents: diazide **1** (1.6 g, 1 mmol), diethyl (2-amino-2-thioxoethyl)phosphonate (**11**) (2.7 g, 1.3 mmol), and 53 mg of Na (2.3 mmol) in 20 mL of EtOH. The reaction mixture was further refluxed for 3 h to give, after the usual working up the phosphonate **13**.

**Diethyl 8-amino-8-mercapto-7,8-dihydroimidazo[1,2-*f*]tetrazolo[1,5-*b*]pyridazin-7-ylphosphonate (13):** Yield 2.3 g (68%); mp 194–196 °C (from $CHCl_3$); $^1$H NMR (500.7 MHz, $CDCl_3$) $\delta_H$ 1.37 (dt, $^3J_{H\text{-}H}$ = 6.5 Hz, $^4J_{P\text{-}H}$ = 4.6 Hz, 2× 3H, 2$H_3$CCOP), 2.17 (br, 1H, *H*S), 4.22 (dq, $^3J_{H\text{-}H}$ = 6.5 Hz, $^3J_{P\text{-}H}$ = 6.1 Hz, 2 × 2H, 2$H_2$COP), 5.36 (d, $^2J_{P\text{-}H}$ = 16.6, 1H, *H*C-P), 6.24 (br, 1H, $H^A$N), 7.11, 8.23 (2d, $^3J_{H\text{-}H}$ = 9.2 Hz, 2 × 1H, 2*H*C(4,5)-pyridazine), 9.18 (br, 1H, $H^B$N); $^{13}$C NMR (125.4 MHz, $CDCl_3$) $\delta_C$ 149.4 (d, $^3J_{P\text{-}C}$ = 7.4 Hz, *C*(2)-imidazole), 146.8 (*C*-tetrazole), 125.1, 124.8 (*C*(4,5)pyridazine), 93.6 (d, $^2J_{P\text{-}C}$ = 16.8 Hz, *C*(5)imidazole), 69.4 (d, $^1J_{P\text{-}C}$ = 178.4 Hz, *C*-P), 62.4 (d, $^2J_{P\text{-}C}$ = 10.3 Hz, $C$H$_2$OP), 16.4 (d, $^3J_{P\text{-}C}$ = 7.5 Hz, $C$H$_3$COP); $^{31}$P NMR (200.7 MHz, $CDCl_3$) $\delta_P$ 27.6; EI-MS: in *m*/*z* (%): 344 $[M - 1]^+$, 316 (24) $[M - 29 (H + N_2)]^+$, 302 (38) $[M – 43 (H + N_3)]^+$, 285 (55) $[M - 60 (H + N_3 + NH_3]^+$, 148 (100) $[M - 197 (H + N_3 + NH_3 + P(O)(OEt)_2)]^+$, 137 (55) ($P(O)(OEt)_2$), 78 (74) (pyridazine); $\nu_{max}$/cm$^{-1}$: 3373–3347 ($NH_2$), 1597 (C=N), 1223 (P=O, bonded), 1166 (tetrazole), 1148 (P-O-C); Anal. calcd for $C_{10}H_{16}N_7O_3PS$ (345.3): C, 34.78; H, 4.67; N, 28.39; P, 8.97; S, 9.29. Found: C, 34.73; H, 4.73; N, 28.45; P, 8.92; S, 9.35.

**Reaction of 1 with 14:** Reagents: diazide **1** (1.6 g, 1 mmol), tetraethyl 1,1-methylbisphosphonate (**14**, 3.7 g, 1.3 mmol) and (53 mg of Na, 2.3 mmol) in 20 mL of EtOH. The reaction mixture was refluxed for 6 h to give, after the usual working up, the methylenebisphosphonate **15**.

**(*z*)-Tetraethyl (tetrazolo[1,5-*b*]pyridazin-6(5*H*)-ylideneamino)methylenediphosphonate (15):** Yield 3.1 g (73%); mp 184–186 °C (from EtOH); $^1$H NMR (500.7 MHz, $CDCl_3$) $\delta_H$, 1.12, 1.28 (2dt, $^3J_{H\text{-}H}$ = 6.7 Hz, $^4J_{P\text{-}H}$ = 4.6 Hz, 2 × 6H, 4$H_3$CCOP), 4.22 (t, $^2J_{P\text{-}H}$ = 18.8 Hz, 1H, *H*C-$P_2$), 4.55, 4.77 (2dq, $^3J_{H\text{-}H}$ = 6.7 Hz, $^3J_{P\text{-}H}$ = 7.2 Hz, 2× 4H, 4$H_2$COP), 7.12, 8.13 (2d, $^3J_{H\text{-}H}$ = 9.2 Hz, 2× 1H,

2$H$C(5,4)-pyridazine), 8.69 (s (br), 1H, $H$N); $^{13}$C NMR (125.4 MHz, $CDCl_3$): $\delta_C$, ppm: 173.3 (t, $^3J_{PC}$ = 7.6 Hz, $C$=N), 139.9 ($C$-tetrazole), 127.2, 126.7 ($C$(4,5)-pyridazine), 61.2 (d, $^2J_{P\text{-}C}$ = 10.4 Hz, $C$H$_2$OP), 52.1 (t, $^1J_{P\text{-}C}$ = 184.4 Hz, $C$-P$_2$), 15.4 (d, $^3J_{P\text{-}C}$ = 7.6 Hz, $C$H$_3$COP); $^{31}$P NMR (200.7 MHz, $CDCl_3$) $\delta_P$ 24.8, 25.6 (2d, $^2J_{P\text{-}P}$ = 28.4 Hz); EI-MS: in $m/z$ (%): 421 (72) $[M - 1]^+$, 393 (40) $[M - 29 (H + N_2)]^+$, 379 (76) $[M - 43 (H + N_3)]^+$, 137 (76) (P(O)(OEt)$_2$), 92 (100) [M − 330 (H + $N_3$ + HC[P(O)(OEt)$_2$]$_2$)]$^+$, 78 (66) (pyridazine); $\nu_{max}$/cm$^{-1}$: 3334 (NH), 1608 (C=N), 1262 (P=O, free), 1226 (P=O, bonded), 1182 (tetrazole), 1123, 1074 (2P-O-C); Anal. calcd for $C_{13}H_{24}N_6O_6P_2$ (422.3): C, 36.97; H, 5.73; N, 19.90; P, 14.67; found: C, 37.01; H, 5.69; N, 19.86; P, 14.63.

**Reaction of 1 with 16**, and **18:** Reagents: diazide **1** (1.6 g, 1 mmol), diethyl vinylphosphonate (**16**) (2.1 g, 1.3 mmol) or diethyl 2-methylallylphosphonate (**18**) (2.4 g, 1.3 mmol), and 53 mg of Na (2.3 mmol) in 20 mL EtOH. The reaction mixture was further refluxed for ca. 4 h (TLC) to give, after the usual working up, the phosphonate **17** or **19**, respectively.

**Diethyl imidazo[1,2-*f*]tetrazolo[1,5-*b*]pyridazin-8-ylphosphonate (17):** Yield 2.2 g (76%); mp 122–124 °C (from $CH_2Cl_2$); $^1$H NMR (500.7 MHz, $CDCl_3$) $\delta_H$ 1.32 (dt, $^3J_{H\text{-}H}$ = 6.6 Hz, $^4J_{P\text{-}H}$ = 4.6 Hz, 2× 3H, 2$H_3$CCOP), 4.19 (dq, $^3J_{H\text{-}H}$ = 6.6 Hz, $^3J_{P\text{-}H}$ = 5.7 Hz, 2× 2H, 2$H_2$COP), 5.65 (d, $^3J_{P\text{-}H}$ = 7.6, 1H, $H$(4)-imidazole), 7.23, 8.14 (2d, $^3J_{H\text{-}H}$ = 9.1 Hz, 2× 1H, 2$H$ (4,5)-pyridazine); $^{13}$C NMR (125.4 MHz, $CDCl_3$) $\delta_C$ 151.3 (d, $^2J_{P\text{-}C}$ = 10.5 Hz, $C$(4)-imidazole), 140.3 (d, $^3J_{P\text{-}C}$ = 7.4 Hz, $C$(2)-imidazole), 131.5 ($C$-tetrazole), 128.8 (d, $^1J_{P\text{-}C}$ = 212.4 Hz, $C$-P), 125.2, 118.2 ($C$(4,5)-pyridazine), 63.5 (d, $^2J_{P\text{-}C}$ = 10.5 Hz, $C$H$_2$OP), 16.5 (d, $^3J_{P\text{-}C}$ = 7.5 Hz, $C$H$_3$COP); $^{31}$P NMR (200.7 MHz, $CDCl_3$) $\delta_P$ 27.8; EI-MS: in $m/z$ (%): 268 (15) $[M - 28 (N_2)]^+$, 254 (49) $[M - 42 (N_3)]^+$, 117 (100) [M − 179 ($N_3$ + P(O)(OEt)$_2$)]$^+$, 137 (48) (P(O)(OEt)$_2$), 78 (72) (pyridazine); $\nu_{max}$/cm$^{-1}$: 1610 (C=N), 1258 (P=O), 1180 (tetrazole),1021 (P-O-C); Anal. calcd for $C_{10}H_{13}N_6O_3P$ (296.2): C, 40.55; H, 4.42; N, 28.37; P, 10.46; found: C, 40.51; H, 4.38; N, 28.34; P, 10.42.

**Diethyl 8,8-dimethyl-7,8-dihydroimidazo[1,2-*f*]tetrazolo[1,5-*b*]pyridazin-7-ylphosphonate (19):** Yield 2.4 g (74 %); mp: 143–145 °C (from cyclohexane); $^1$H NMR (500.7 MHz, $CDCl_3$) $\delta_H$ 1.13 (dt, $^3J_{H\text{-}H}$ = 6.7 Hz, $^4J_{P\text{-}H}$ = 4.9 Hz, 2 × 3H, 2$H_3$CCOP), 1.73-1.87 (m, 2 × 3H, 2$H_3$C), 3.86

(dq, $^3J_{H\text{-}H}$ = 6.7 Hz, $^3J_{P\text{-}H}$ = 5.8 Hz, 2 × 2H, 2$H_2$COP), 5.25 (d, $^2J_{P\text{-}H}$ = 20.2, 1H, *H*C-P), 7.21, 8.23 (2d, $^3J_{H\text{-}H}$ = 9.1 Hz, 2 × 1H, 2*H*C(4,5)-pyridazine); $^{13}$C NMR (125.4 MHz, $CDCl_3$) $\delta_C$ 152.5 (d, $^3J_{P\text{-}C}$ = 8.7 Hz, *C*(2)-imidazole), 142.1 (*C*-tetrazole), 125.4, 123 (*C*(4,5)-pyridazine), 64.5 (d, $^1J_{P\text{-}C}$ = 188.4 Hz, *C*-P), 62.5 (d, $^2J_{P\text{-}C}$ = 10.7 Hz, $CH_2$OP), 54.8 (d, $^2J_{P\text{-}C}$ = 11.3 Hz, *C*(5)-$Me_2$), 24.4, 21.5 (2d, $^3J_{P\text{-}C}$ = 7.8, 7.6 Hz, 2$CH_3$), 16.8 (d, $^3J_{P\text{-}C}$ = 7.4 Hz, $CH_3$COP); $^{31}$P NMR (200.7 MHz, $CDCl_3$) $\delta_P$ 27.8; EI-MS: in *m*/*z* (%): 325 (18) $[M - 1]^+$, 297 (35) $[M - 29 (H + N_2)]^+$, 283 (49) $[M - 43 (H + N_3)]^+$, 253 (34) $[M - 73 (H + N_3 + 2Me)]^+$, 137 (55) $(P(O)(OEt)_2)$, 116 (100) $[M - 210 (H + N_3 + 2Me + P(O)(OEt)_2)]^+$, 78 (71) (pyridazine); $\nu_{max}/cm^{-1}$: 1605 (C=N), 1256 (P=O), 1178 (tetrazole), 1095 (P-O-C); Anal. calcd for $C_{12}H_{19}N_6O_3P$ (326.29): C, 44.17; H, 5.87; N, 25.76; P, 9.49; found: C, 44.26, H, 5.94; N, 25.69; P, 9.43.

## Anti-nociceptive evaluation

***p*-Benzoquinone (p-BQ)-induced writhing test:** Sixty minutes after subcutaneous administration of the test samples: **1**, **4**, **7**, **10a**, **10b**, **13**, **15**, **17**, and **19** dosing vehicle and ibuprofen (50 mg/kg), the mice were intraperitoneally injected 2.5% (v/v) *p*-BQ solution in distilled water. Then, the mice were kept individually for observation and the total number of abdominal contraction (writhing movements) was counted for the next 15 min, starting from the fifth minute after the *p*-BQ injection. The results of the analgesic capacity of the test compounds on *p*-BQ-induced abdominal constriction test were displayed in Table 1. The anti-nociceptive capacity was expressed as the percentage change compared to writhing controls.

$$\% \text{ capacity} = [(N_c - N_t) / N_c] \times 100$$

$N_c$ and $N_t$ are the total number of writhing movements at 0 time and after 15 min.

## Anti-inflammatory screening

**Carrageenan-induced hind paw edema test:** The anti-inflammatory evaluation for new compounds, **4**, **7**, **10a**, **10b**, **13**, **15**, **17**, **19,** and the substrate **1**, was in vivo determined by carrageenan-induced hind paw edema standard method [2,3]. Wister albino rats of either sex (pregnant female animals were excluded) weighing 100-150 g were divided into 10 groups of 6 rats each. The rats were fed a standard pellet diet and given water ad-libitum and were kept in rooms maintained under proper temperature (25–30 °C), ventilation, and hygienic conditions.

They were exposed to 12 h each of light and darkness. Regulations concerning the care and use of laboratory animals were considered during the pharmacological screening. Carrageenan paw edema was induced by subcutaneous injection of 1% solution of carrageenan in saline (0.1 mL/rat) in the right hind paw of rats. Administration of **1**, **4**, **7**, **10a**, **10b**, **13**, **15**, **17**, **19** and indomethacin (**A**) dissolved in DMSO, in a dose of 50 mg/kg body weight was given surgically in the left hind paw of rats 1 h before induction of inflammation. The control group was given the DMSO solution only. Paw edema was measured over each 90 min during 6 h after induction of inflammation. The difference in footpad thickness between the right and left foot was measured with a pair of dial thickness gauge calipers. Mean values of treated groups were compared with mean values of a control group. The anti-inflammation activity was expressed as percent inhibition of edema volume in treated animals in comparison with the control group. The results were displayed in Table 1.

$$\%\ \text{inhibition of edema} = [(V_c - V_t)/V_c] \times 100$$

Where $V_c$ and $V_t$ are the volumes of edema for the control and tested substance-treated animal groups, respectively. Potency of the tested compounds was calculated regarding indomethacin, reference standard, according to the following equation:

$$\%\ \text{Activity} = \frac{\%\ \text{edema inhibition of tested-compound treated group}}{\%\ \text{edema inhibition of indomethacin treated group}} \times 100$$

## References


1. Shin, M. S.; Kang, Y. J.; Chung, H. A.; Park, J. W.; Kweon, D. H.; Lee, W. S.; Yoon, Y. J.; Khim, S. K. *J. Heterocycl. Chem.* **1999,** *36*, 1135–1142. doi:10.1002/jhet.5570360504
2. Okun, R.; Liddon, S. C.; Lasagna, L. *J. Pharmacol. Exp. Ther.* **1963,** *139*, 107–109.
3. Winter, C. A.; Risley, E. A.; Nuss, G. W. *Proc. Soc. Exp. Biol. Med.* **1962,** *111*, 544–547.
